# Effect of the Instant Controlled Pressure Drop Technology in Cardamom (*Elettaria cardamomum*) Essential Oil Extraction and Antioxidant Activity

**DOI:** 10.3390/molecules27113433

**Published:** 2022-05-26

**Authors:** Giselle Dení Teresa-Martínez, Anaberta Cardador-Martínez, Carmen Téllez-Pérez, Karim Allaf, Cristian Jiménez-Martínez, Maritza Alonzo-Macías

**Affiliations:** 1 Escuela de Ingeniería y Ciencias, Tecnologico de Monterrey, Epigmenio González 500, Fracc. San Pablo, Queretaro C.P. 76130, Mexico; gis_teresa20@outlook.com (G.D.T.-M.); mcardador@tec.mx (A.C.-M.); ctellezperez@gmail.com (C.T.-P.); 2 Intensification of Transfer Phenomena on Industrial Eco-Processes, Laboratory of Engineering Science for Environment, University of La Rochelle, LaSIE—UMR-CNRS 7356, 17042 La Rochelle, France; kallaf@univ-lr.fr; 3 Escuela Nacional de Ciencias Biológicas, Instituto Politécnico Nacional, Av. Wilfrido Massieu Esq. Manuel Stampa, Gustavo A. Madero, Ciudad de Mexico C.P. 11340, Mexico

**Keywords:** cardamom, essential oil, antioxidants, instant controlled pressure drop, ABTS, DPPH, hydrodistillation

## Abstract

Green cardamom (*Elettaria cardamomum*) is an outspread spice native to Asia, which is well appreciated for its sensory characteristics, delicate aroma, and unique taste. Currently, the main cardamom extracts are essential oils (EOs), and regarding current market tendencies, this market is in high growth. For this reason, technologies such as the instant controlled pressure drop (DIC) have been applied to reach higher yields and better quality of EO. Then, this study explores the impact of DIC as a pretreatment before hydrodistillation (HD) on the EO yield and their antioxidant activity. Obtained results showed that the coupling of DIC-HD increased the yield of essential oil and also had a positive impact on their antioxidant capacity. The EO yield of DIC-HD (140 °C and 30 s) was 4.43% vs. 2.52% for control; the AOX of DIC-HD (165 °C and 30 s) was 86% inhibition vs. 57.02% for control, and the TEAC of DIC-HD (140 °C and 30 s) was 1.44 uMTE/g EO vs. 13.66 uMTE/g EO.

## 1. Introduction

Green cardamom (*Elettaria cardamomum*) is a widespread spice native to the Asian continent which is often referred to as the queen of spices due to its very pleasant, mild aroma and taste that is appreciated for its organoleptic characteristics for culinary purposes or its properties in traditional medicine [1,2]. Initially, cardamom cultivation was solely placed in India by British planters. Nowadays, cardamom is grown in India, Guatemala, Sri Lanka, Nepal, Indonesia, Costa Rica, Mexico, and Tanzania; being Guatemala the major producer, followed by India [3,4]. 

Since ancient times, cardamom has long been used in India [5]. Assyrian doctors and chemists were known to apply many herbs, including cardamom, for medicinal purposes [5,6]. Historical records of cardamom usage can be found along with Greece and Rome, where spices were a symbol of luxury and status. Their importance was such that they were included in social and religious ceremonies [6]. 

Nowadays, cardamom is still used as a part of cosmetic formulations and an ingredient in the food and pharmaceutical industries. The main cardamom extracts are essential oils (EOs). Currently, the importance of EOs in the industry has grown due to the consumers’ inclination to use natural products, i.e., the usage of EO in the industry goes from food and beverage to cosmetics and aromatherapy [7]. In 2018 the demand for essential oil was 226.8 kilotons, and the global market for essential oil is expected to grow by 8.6% from 2019 to 2025 [7]. EO has been proven to have many beneficial properties, such as antioxidant activity (AOX), antispasmodic, antimicrobial, and carminative, among many others; being these actions directly influenced by its specific chemical composition [7,8,9].

Traditionally essential oil extraction has been achieved via hydrodistillation. This technique is based on the principle that, at boiling temperature, the combined vapor pressures equal the atmospheric pressure. The charged steam rises and encounters a narrow tube cooled by an outside source (cold water or antifreeze). Afterward, the steam is condensed and collected in a vessel; hence, since the essential oil is less dense, it moves at the top while the water goes down [10,11]. Some advantages of this procedure are the low cost of the equipment and its simplicity. On the other hand, it also has disadvantages, such as it can consume a lot of energy and the processing time is often long, which could trigger negative chemical changes on the EO. Regarding energy consumption, in the study conducted by Allaf et al. [12] on orange peels, the energy balances of HD vs. the instant controlled pressure drop technology (DIC) point out that above a certain amount of extraction efficiency, the energy consumption significantly decreases by using the DIC technology. 

The instant controlled pressure drop technology (DIC) was first created to intensify the drying unit operation by texturing bioproducts. Shortly after, its applications were spread to other pharmaceuticals, food, and cosmetic unit operations such as food decontamination, phytochemicals extraction, and EOs extraction [13]. DIC is a thermo-mechanical technique that subjects the sample to a high-pressure saturated steam (0.1–1.0 MPa) for a brief period (some seconds), followed by an instant pressure drop close to a vacuum (30 mbar) [14]. The instant pressure drop towards the vacuum causes the autovaporization of the water, the swelling of the matrix, and a possible breakdown of the cell wall. Then, materials treated by DIC can highly increase the extraction kinetics by improving solvent diffusivity within the solid and enhancing mass transfer [14,15]. Due to these characteristics, it could be suggested that by coupling DIC technology to HD, the extraction efficiency of EO and their biological activities could be improved.

To the best of our knowledge, there are no previous studies regarding DIC technology as a texturing pretreatment of cardamom seeds before hydrodistillation. Then, this comparative study explores the impacts on the essential oil yield extraction and the antioxidant scavenging capacity of EO obtained by only HD and by coupling DIC to HD.

## 2. Results and Discussion

### 2.1. Seed Structure and Moisture Content Changes

**Figure 1:**
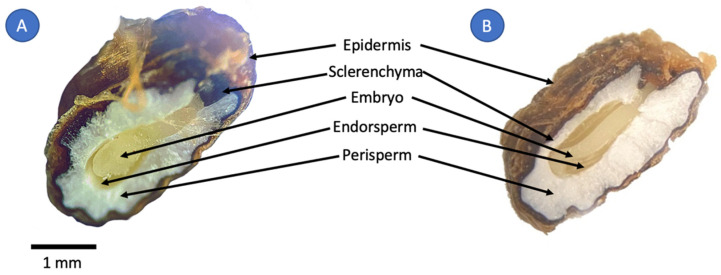
Cardamom seed morphology: (**A**) Untreated cardamom seed (**B**) DIC-treated cardamom seed (140 °C and 30 s).

Stereo microscopy was used to monitor the morphological changes in the seed. Figure 1 denotes a side-by-side comparison between an untreated and a DIC-treated seed. As it can be observed in Figure 1A, in the untreated seed, the perisperm layer appears compacted and arranged crystal-like. Conversely, in the DIC-treated seed, the same layer appears bloated and less compact (Figure 1B).

These changes could be attributed to both main stages of DIC treatment: the steam condensation occurred during the saturated steam injection into the reactor (which increased the initial moisture content of the seed) and also to the autovaporization of seed water (which could trigger a cardamon seed expansion). In fact, as shown in Table 1, while the control seeds performed average moisture values of 5.1% d.b, the DIC-treated seeds performed moisture contents between 10.5 to 11.3% d.b.

Moreover, although there was an increase in moisture content after DIC treatment, these values fall into the specifications of the American Spice Trade Association (ASTA), which suggests a moisture content not greater than 12% [16,17]. 

Then, this study suggests that the DIC treatment allowed a rapid increase in moisture content, which subsequently engendered a cardamon seed expansion due to autovaporization, which positively impacts both the EO yield and the antioxidant scavenging capacity. This effect has been previously acknowledged on different matrices such as orange peel (*Citrus sinensis*), Myrtle leaves (*Myrtus communis* L.), Hyssop (*Hyssopus officinalis* L.), among others [14,18,19,20,21]. Moreover, it is also worth bearing in mind that the rate of expansion of the matrix is closely related to the working parameters of DIC (saturated steam pressure directly linked to the temperature, processing time, and, in some cases, the number of cycles), and the biological matrix composition (initial moisture content, and chemical composition) [21].

### 2.2. Essential Oil Yield

**Figure 2:**
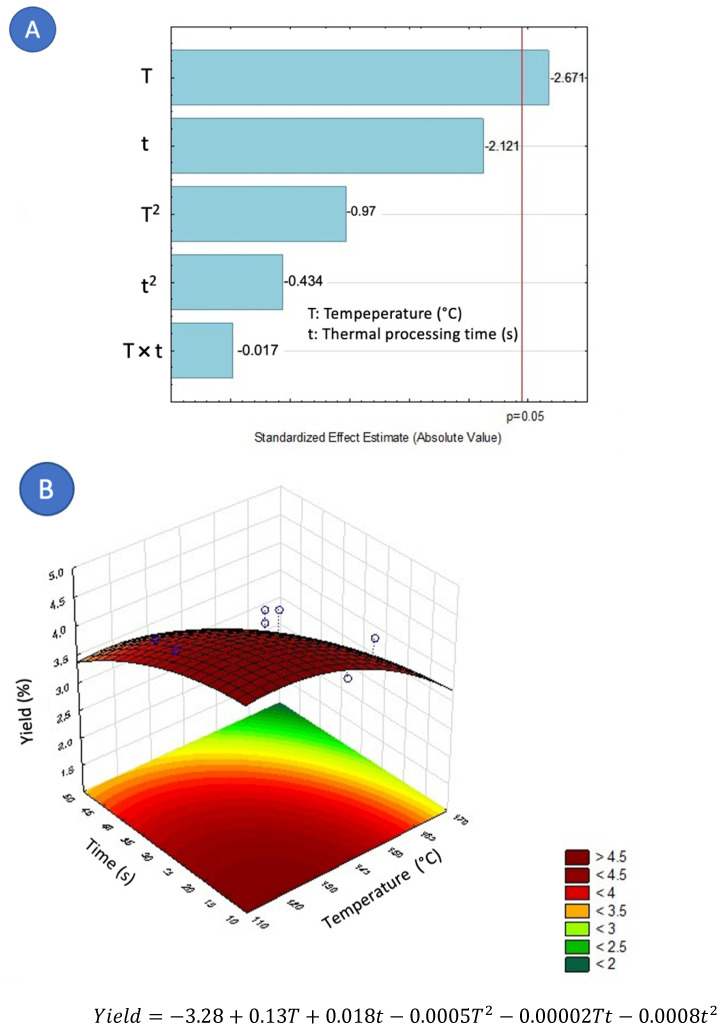
Effect of steam processing temperature “T” (°C) and thermal processing time “t” (s) on yield (%) of EO: (**A**) Pareto chart (**B**) Surface response.

HD and DIC-HD extracted oils presented a clear appearance and a pleasant aroma. On the other hand, as can be observed in Table 1, the highest extraction percentages were 4.43%, 4.40%, and 4.39% from DIC 12 (140 °C, 30 s, 0.36 MPa), DIC 11 (140 °C, 15 s, 0.36 MPa), and DIC 8 (122 °C, 41 s, 0.21 MPA). In contrast, the lowest extraction yields were provided by the control, DIC 1 (165 °C, 30 s, 0.7 MPa) and DIC 2 (140 °C, 45 s, 0.36 MPa), with yields of 2.52%, 2.54%, 2.56%, respectively. As can be remarked in Figure 2A, the Pareto chart showed that the factor affecting the yield was the temperature, being this negative; the higher the DIC treatment temperature, the lower the EO yield. Furthermore, according to the response surface graph (Figure 2B), the oil yield extraction could be enhanced under a temperature range between 110 and 140 °C and a treatment time between 10 to 20 s.

Essential oils have been a part of human life since ancient times. Cardamom essential oil has been used as an important part of folk medicine, rituals, cosmetics, and perfumes [22]. Due to its great importance and the economic significance of cardamom, any increase in EO yield is welcomed. As shown in Table 1, the highest yield was obtained in the sample DIC 12 (140 °C, 30 s, 0.36 MPa) with a 4.44%. Studies provide a wide range of values among the same matrices. Most of these studies have used cardamom varieties from different geographic locations, where Guatemalan samples gave 3.74% [23], Indian samples gave 3.1% [24], and 1% from Turkey samples [25]. These studies were carried out by extracting the EO via hydrodistillation in the Clevenger apparatus, the same as this study. By comparing EO yields from only HD to DIC-HD, the percentage of EO extracted from treated samples was overall higher than those of the untreated. It is worth noting that the EO yield is a multifactorial response and can be influenced by multiple factors such as plant variety, growing conditions, harvesting conditions, post-harvest processing (i.e., drying method), geographic area, etc. The study conducted by Allaf et al. [21] showed a positive impact on the extraction yield, such as the optimization of EO extraction, from orange peels, where EO from hydrodistillation extraction was achieved in 4 h to obtain a yield of 1.97 mg/g db, while DIC optimized treatment (0.6 MPa, 11 cycles of 11 s) performing a yield of 16.57 mg/g db. For cardamon seeds, the increase in the essential oil yield can also be attributed to the increase in the porosity in the epidermis.

### 2.3. DPPH Free Radical Scavenging Capacity

**Figure 3:**
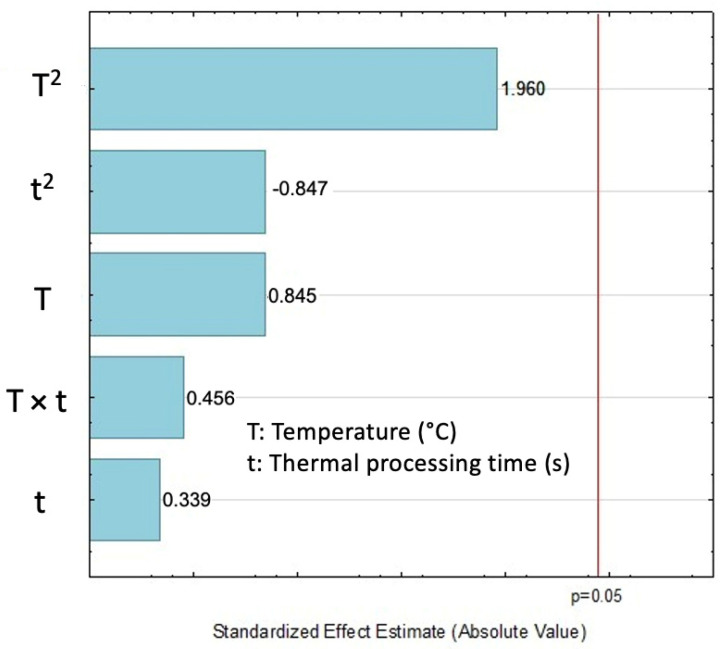
Effect of steam processing temperature “T” (°C) and thermal processing time: “t” (s) on AOX (%) of EO: Pareto chart.

The results of the DPPH assessment expressed as antioxidant capacity (AOX) can be seen in Table 1. The strongest antioxidant activity was obtained under DIC 3 (140 °C, 30 s, 0.36 MPa) with a 68%. The lower AOX was obtained under DIC 1 (165 °C, 30 s, 0.7 MPa), DIC 8 (122°C, 41 s, 0.21 MPa), and DIC 12 (140 °C, 30 s, 0.36 MPa) treatments with 62.28%, 65.38% and 65.38% respectively. The Pareto chart (Figure 3) showed that neither temperature nor thermal processing time could explain the antioxidant activity changes between the selected DIC studied parameters. 

By comparing the AOX of essential oil extracted from treated seeds to control, it can be shown an increase in AOX; while DIC 3 (140 °C, 30 s, 0.36 MPa) performed an AOX of 68%, the AOX of control performed 57%. Regarding other studies, the maximum percentage reached has been 70% [26,27,28]. Evidence of the positive impact of DIC treatment on antioxidant compounds is provided in the study conducted by Mounir et al. [29] in expanded granule powder of apple and onion. In that study, quercetin extraction was increased by 6.8 times compared to untreated samples due to DIC treatment. Another example of the beneficial effects of DIC treatment on antioxidant activity is provided by Alonzo-Macías et al. [30], who evaluated the impact of different drying methods on the total phenol, total flavonoid, total anthocyanin content, and antiradical activity by using DPPH of strawberries. The results showed an average increase of 9.5% in the antioxidant capacity of DIC-treated samples. Similarly, Namir et al. [31] observed an increase of 1.5 times in AOX of DIC-treated vs. untreated Cactus pear peel snacks samples. DIC preserves the quality of secondary metabolites thanks to the water’s autovaporization that guarantees rapid cooling, which prevents the thermal degradation of sensitive compounds.

### 2.4. ABTS Trolox Equivalent Antioxidant Capacity Determination (TEAC)

**Figure 4:**
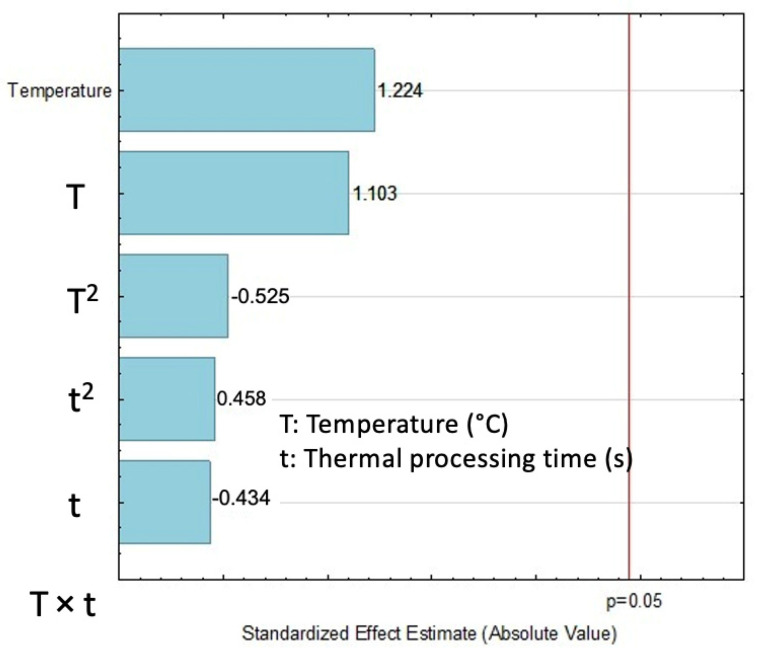
Effect of steam processing temperature “T” (°C) and thermal processing time “t” (s) on TEAC (uMTE/g EO) of EO: Pareto chart.

Contrary to yield and AOX, in which a maximum value is desirable, the least TEAC means a better antioxidant capacity in this study. The summary of TEAC for each sample is shown in Table 1. The better performers for TEAC were found by DIC 6 (130 °C, 30 s, 0.36 MPa) with 1.99 uMTE/g EO and by DIC 3 (140 °C, 30 s, 0.36 MPa) with 2.04 uMTE/g EO. On the other hand, the untreated seed (control) gave 13.66 uMTE/g EO, indicating low antioxidant capacity. In Figure 4, the Pareto chart showed that the TEAC was not influenced by temperature or thermal processing time under the selected DIC studied parameters. 

According to recent research, the results of ABTS assays in non-treated cardamom are negligible [32,33]. Interestingly, the ABTS results were significant for all samples in this research, presumably due to the DIC treatment. A study supporting this affirmation is the conclusion reached by Tellez-Perez et al. [34], who observed TEAC enhancement by as much as 2.89 (depending on the DIC conditions). In our study, the best TEAC was obtained at lower time and temperature values; in contrast with poblano pepper, they reached the highest TEAC values of both steam pressure and holding time. An interesting approach would be to test the effect of more variables such as the initial moisture content, the treatment time, the temperature, and the number of DIC cycles, to have a complete picture of their interactions and how they affect the response variables.

## 3. Materials and Methods

### 3.1. Materials

Cardamom seeds were obtained from finca Argovia in Tapachula Chiapas, México. All solvents used were HPLC grade (Sigma-Aldrich, St. Louis, MO, USA.).

### 3.2. Methods

#### 3.2.1. Moisture Content

The moisture content of the seeds was determined by a dynamic method using a laboratory air dryer (Binder FD 23). Two grams of seed were placed in the dryer at a temperature of 105 °C for 24 h. 

#### 3.2.2. Experimental Design and Statistical Analysis

Response surface methodology was accomplished with a central composite design which led to 12 experiments carried out with four center points. The response variables were EO Yield (%), Antioxidant capacity (AOX in %), and Trolox Equivalent Antioxidant Capacity (TEAC in uMTE/g EO), and the considered factors were Saturated Steam Temperature (°C) and Thermal Processing Time (s). Table 2 shows the experimental design. 

Statistica Software 2017 (TIBCO Software Inc., Palo Alto, CA, USA) was used for the statistical analysis. For the experimental design of DIC treatment, statistical analysis was performed through the Pareto chart and the surface response methodology. The Pareto chart was used to identify the impact of variables on the responses. The vertical line in the Pareto chart determines the statistically significant effects at the 95% confidence level. Saturated steam temperature (°C) and thermal processing time (s) were studied as independent variables. The studied response variables were: EO Yield (%), AOX (%), and TEAC (μM TE/g EO).

#### 3.2.3. DIC Treatment

**Figure 5:**
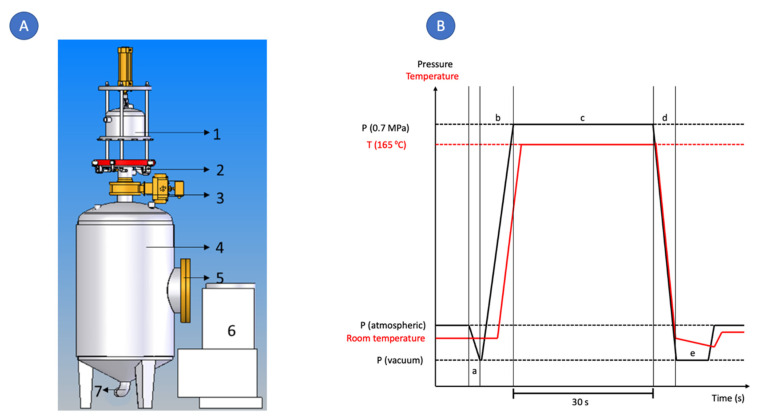
(**A**) Schematic representation of a DIC laboratory equipment (1) Double-walled bell, (2) Crown, (3) Decompression valve, (4) Vacuum tank, (5) Manhole, (6) Vacuum pump, (7) Condensates extraction pipe. (**B**) Schematic of a DIC processing cycle (a): Establishment of the vacuum; (b): injection of steam; (c) pressure maintenance during established thermal processing time; (d): instant controlled pressure drop towards the vacuum; (e): stabilization at atmospheric pressure.

The DIC treatment of Cardamon seeds was carried out in four steps. Figure 5A show the schematic diagram of a DIC apparatus, and Figure 5B shows the schema of a DIC processing cycle. Firstly, 100 g of seeds were placed into the DIC reactor, in which a vacuum of 30 mbar was established (Figure 5(Ba). Secondly, as can be observed in Figure 5(Bb,Bc), saturated steam was injected into the reactor until the selected study saturated steam temperature was reached (being from 0.17 up to 0.7 MPa), and this was maintained for a short time (being from 15 to 45 s). Thirdly, samples were subjected to an instant controlled pressure drop (ΔP/Δt > 0.5 MPa.s^−1^) towards vacuum (30 mbar) (Figure 5(Bd)); in fact, this pressure drop causes the autovaporization of the water and the swelling of the matrix. Finally, the pressure was released toward the atmospheric pressure (Figure 5(Be)), and cardamon seeds were recovered. In this study, the used DIC equipment was a LAB DIC 0.1 model (ABCAR-DIC Process, La Rochelle, France). The impact of DIC treatment on morphology, essential oil yield, and radical scavenging activity by DPPH and TEAC was evaluated using a surface response design (Table 2). After DIC treatment, cardamom seeds were stored at −80 °C until further analysis.

#### 3.2.4. Stereo Microscopy

Cardamom seeds morphology was evaluated by stereo microscopy using a Rossbach Kyowa (Mexico) stereo microscope with series number 751010. A cross-section cut was taken of both untreated seed and a treated seed to observe changes in structure.

#### 3.2.5. Essential Oil Extraction via Hydrodistillation

**Figure 6:**
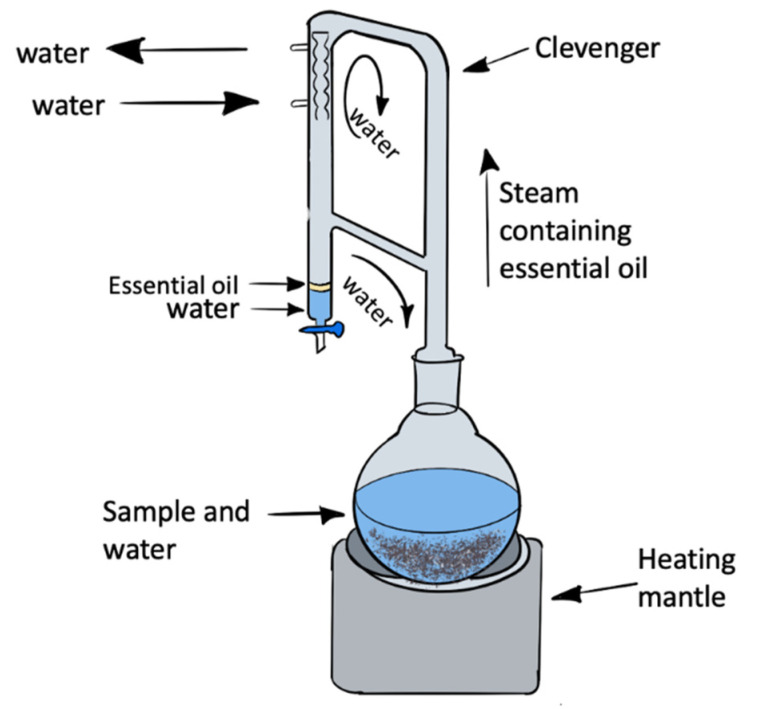
Clevenger apparatus for hydrodistillation (This figure was made with Procreate 5.2).

For this study, 50 g of grounded cardamom were mixed with 600 mL of distilled water in a Clevenger-type apparatus and distilled for six hours (Figure 6). The essential oil was dried over anhydrous sodium sulfate and stored [11,35] until analysis. The yield was calculated as grams of oil per 100 g of seed (dry basis).

#### 3.2.6. Radical Scavenging Activity by DPPH

The DPPH analysis by Fukumoto et al. [36] is based on the radical unpaired electron yield to an antioxidant substance; DPPH is demoted from blue-purple color to light yellow. For this assay, a stock solution of DPPH 125 μM was prepared. For the assay, 20 μL of samples and 200 μL of DPPH were added to a well in a 96-microwell plate and mixed; analysis was carried out by triplicate. The plaque was stored in the dark for 90 min at room temperature. The concentration of essential oil in the sample was 0.19 g/mL. Absorbance readings were taken at 520 nm using an xMark™ Microplate Absorbance Spectrophotometer (Bio-Rad, Hercules, CA, USA). Scavenging was expressed as percent DPPH discoloration.

#### 3.2.7. Trolox Equivalent Antioxidant Capacity (TEAC)

Trolox equivalent antioxidant capacity is based on the comparison between the antioxidant capacity to cleave the radical cation of ABTS and Trolox, as described by Re et al. [37]. ABTS stock solution by reacting 7 mMol/L and 2.45 mMol/L of potassium persulfate after incubation in the dark for 16 h. The stock solution was then diluted in ethanol to an absorbance of 0.8 ± 0.1 at 734 nm. Trolox standard solutions were prepared in methanol from 0 to 700 μmol/L and assayed under the same conditions. In each well of a 96-microplate, 200 μL of reagent and 20 μL of the sample were added and incubated for 6 min with constant agitation. Each sample was assessed in triplicate. The concentration of essential oil in the sample was 0.62 mg/mL. The readings were performed at 734 nm using xMark™ Microplate Absorbance Spectrophotometer (Bio-Rad, Hercules, CA, USA). Trolox standard solutions were prepared in methanol from 0 to 700 μmol/L and assayed under the same conditions. The Trolox equivalent antioxidant capacity (TEAC) was calculated based on the Trolox calibration curve and reported as the μM/L needed for Trolox to decolorate at the corresponding concentration.

## 4. Conclusions

Traditionally essential oil extraction of cardamon seeds has been achieved via hydrodistillation (HD); however, this technique presents some drawbacks as high energy consumption and possible thermal degradation of EO due to long extraction kinetics. For these reasons, this study has evaluated the effect of coupling DIC technology to HD on EO’s extraction efficiency and biological activities.

In this respect, results showed a clear improvement in cardamon yield and antioxidant activity by coupling DIC technology to hydrodistillation. DIC-HD (140 °C, 30 s, 0.36 MPa) does show an increase in the EO yield of 1.7 times (4.44% from DIC vs. 2.52% for only HD). In addition to this, DIC-HD essential oils improved antioxidant capacities with HD. Regarding AOX by DPPH, under the selected studied parameters of saturated steam temperature and thermal processing time, all applied DIC treatments allowed for increased the AOX of extracted essential oils. The highest AOX was found under DIC 3 (140 °C, 30 s, 0.36 MPa), being 68%, vs. the control with 57%. Concerning TEAC, the results showed that DIC pretreatment improved the antioxidant activity, being found the best value of TEAC under DIC 6 (130 °C, 30 s, 0.36 MPa) with 1.988 uMTE/g EO; control performed a TEAC value of 13.66 uMTE/g EO, indicating a lower antioxidant capacity.

Regarding the morphological changes in cardamon seeds, it could be suggested that the increase in the essential oil yield and antioxidant activity can be attributed to the increase in the porosity of the matrix; however, more scanning electron microscopy studies are needed to explain the microstructural changes of the seed attributed to the DIC treatment.

Finally, it is necessary to note that more studies are being carried out on the chemical composition of cardamom essential oil to evaluate the effect of DIC and the relationship between its composition and its antioxidant activity.

## Figures and Tables

**Table 1:** Effect of DIC treatment on cardamom seeds moisture content, essential oil yield, and antioxidant capacity.

Sample	Treatment Conditions		Response Variables			
	Steam Processing Temperature (°C)	Thermal Processing Time (s)	Moisture Content (%, d.b.)	Yield (%)	AOX (%)	TEAC (uMTE/g EO)
CONTROL	-	-	5.14	2.52	57.02	13.66
DIC 1	165	30	11.27	2.57	62.28	13.52
DIC 2	140	45	10.90	3.08	66.59	2.17
DIC 3	140	30	11.16	3.60	68.18	2.04
DIC 4	158	41	11.33	3.49	67.20	2.18
DIC 5	158	19	11.04	4.01	66.25	2.15
DIC 6	140	30	10.59	3.85	66.78	1.99
DIC 7	122	19	10.80	4.39	66.68	2.04
DIC 8	122	41	10.50	3.89	65.38	2.11
DIC 9	140	30	10.83	4.22	66.34	2.97
DIC 10	115	30	10.83	4.40	66.44	2.12
DIC 11	140	15	10.74	4.26	66.78	2.38
DIC 12	140	30	10.64	4.44	65.38	2.04

d.b.: dry basis. AOX: antioxidant activity by DPPH expressed as percent discoloration. TEAC: Trolox equivalent antioxidant capacity expressed as μM eq. Trolox.

**Table 2:** Experimental design of the DIC pretreatment parameters.

Treatment	T (°C)	t (s)	T (°C)	t (s)
DIC 1	1	0	165	30
DIC 2	0	1	140	45
DIC 3	0	0	140	30
DIC 4	+α	+α	158	41
DIC 5	+α	−α	158	19
DIC 6	0	0	140	30
DIC 7	−α	−α	122	19
DIC 8	−α	+α	122	41
DIC 9	0	0	140	30
DIC 10	−1	0	115	30
DIC 11	0	−1	140	15
DIC 12	0	0	140	30

T = Saturated steam temperature; t = thermal processing time.
